# Associations of physical activity, sedentary time, sleep quality, and resting EEG with mild depressive symptoms in college students: a cross-sectional study

**DOI:** 10.1186/s12889-025-25221-7

**Published:** 2025-11-24

**Authors:** Xiang Wang, Mi Hu, Jing Wang, Xing Wang

**Affiliations:** 1 Department of physical education, East China University of Political Science and Law, Shanghai, 201620 China; 2 Department of Sports and Health, Shanghai Lixin University of Accounting and Finance, Shanghai, 201620 China; 3 Department of physical education, Shanghai University of Sport, Shanghai, 200438 China

**Keywords:** Mild depressive symptoms, Physical activity, Sedentary time, Sleep, Resting EEG, Correlation

## Abstract

**Background:**

This cross-sectional study aims to describe the characteristics of physical activity, sedentary time, sleep quality, and resting EEG among college students with mild depressive symptoms, and further explore pairwise correlations between behavioral patterns, resting EEG, and mild depressive symptoms.

**Methods:**

This study included 75 college students with mild depressive symptoms (MDS) and 75 college students without depressive symptoms (ND) as research subjects. Physical activity (vigorous physical activity (VPA), moderate physical activity (MPA), and low physical activity (LPA)) and sedentary time（ST） were measured using the International Physical Activity Questionnaire Short Form (IPAQ-SF). Sleep quality was measured using the Pittsburgh Sleep Quality Index (PSQI). Resting EEG power values were collected from subjects in a quiet, eyes-closed state using an electroencephalography (EEG) device.

**Results:**

(1) Characteristic analysis revealed that compared with the ND group, the MDS group exhibited reduced MPA and VPA scores, elevated ST scores, and increased total PSQI scores along with elevated scores across its subdimensions. Their behavioral patterns (Moderate-to-Vigorous Physical Activity (MVPA), Sedentary Behavior (SB), Poor Sleep Quality (PSQ) may have changed, including a decrease in the proportion of MVPA, an increase in the proportion of SB, and an increase in the proportion of PSQ. Analysis of resting EEG revealed increased Alpha2 (α2) band power in the temporal regions (T3 and T5) and increased Beta1 (β1) band power in the frontal region (Fp1) in the MDS group (all *p*_*FDR*_ < 0.01). (2) Exploratory analyses revealed that SB and PSQ behaviors may be positively correlated with mild depressive symptoms, while MVPA may be negatively correlated with mild depressive symptoms. The combination of certain EEG indicators (α2 at T3 and T5, β1 at Fp1) achieved an AUC of 0.659 (95% CI: 0.572 to 0.745, *p* = 0.001) for identifying mild depressive symptoms. Additionally, in the MDS group, sleep efficiency scores showed a negative correlation with Alpha1 (α1) band power at the frontal region (F4) electrode position, sleep disturbance scores showed a negative correlation with theta (θ) band power at the parietal region (P3), hypnotic medication scores showed a positive correlation with beta1 (β1) band power at the frontal region (F3), and total PSQI scores showed a negative correlation with θ band power at the temporal region (T3).

**Conclusion:**

College students with mild depressive symptoms may exhibit altered behavioral patterns and abnormal neural activity in the frontal and temporal regions. Their changed behavioral patterns may correlate with mild depressive symptoms, and recognition models based on certain resting EEG indicators demonstrate preliminary application potential. The association between specific sleep issues and localized EEG activity in this population may provide evidence for further elucidating the mechanistic pathways linking their behavior and brain activity. Future longitudinal studies are recommended to explore causal relationships among these variables.

## Introduction

A number of reports have indicated that college students are vulnerable to depressive symptoms due to the challenges, pressures and increased autonomy they face at this stage of their lives, which is a critical issue [1]. About 18.5% of college students have experienced depressive symptoms in the past 12 months, a significantly higher rate than the 9.6% prevalence among non-college students, with Mild Depressive Symptoms(MDS) being the most common [2]. Prolonged exposure to such high-risk scenarios is prone to exacerbate the symptoms, and generate living and financial burdens [3]. However, due to the relative invisibility of mild depression symptoms, schools and society have not paid enough attention to this issue [4]. In response, National Health Commission of People’s Republic of China recently noted that colleges and universities need to enhance their efforts to prevent and identify early depression, which should be included in student health checkups.

There is a well-established link between behavioural patterns and mental illness. Physical activity, sitting and sleep constitute key daily behaviours that have received significant attention [5, 6]. Studies have found that regular physical activity provides physical, mental and emotional benefits [7]. Conversely, increased sedentary time is related to higher risks of conditions such as cardiovascular disease and depression [8]. Sleep has also been strongly correlated with depressive symptoms in several studies [9]. Behavioural pattern, as a variable factor, have been mentioned in many studies in response to mental illness [10]. For example, public health guidelines recommend engaging in moderate to high-intensity physical activity on a daily basis, which is helpful for resisting depressive symptoms [11]. Sedentary behaviours may have a negative impact on mood regulation and reducing such behaviours has been shown to lower levels of inflammatory factors [12]. Sleep problems, often overlooked by college students, may sometimes precede the onset of depressive symptoms [13]. Improving low sleep quality reduce the risk of mental illness and depression [14]. There may be an interaction between physical activity, sedentary behaviour and sleep. Exploring the combined effects of all three behavioural patterns needs to be a priority for scholars [5, 15]. MDS may lie at the borderline between normalcy and depressive symptoms, and little is known about the behavioural patterns of this population. The correlation between such behavioural patterns and the occurrence of MDS has yet to be validated. Clarification of the behavioural patterns of individuals in the possible transition stage is important for better characterizing this population and developing targeted prevention programmes.

Advances in EEG technology have made it a powerful tool for the non-invasive study of neurological disorders, including depression. As an affective disorder associated with functional changes in the frontal cortex and limbic system, it is feasible to understand the pathogenesis through changes in EEG activity and to assist in the detection of depressive symptoms [16]. Changes in brain structure and function are accompanied by alterations in brain bioelectric signals. Spontaneous EEG signals are widely used to reflect the processing of internal psychological states [17]. It has been suggested that brain activity in depressed patients correlates with the severity of symptoms, with abnormal brain activity reflecting an increase in symptom severity [18]. Depression is associated with increased power in Theta (θ), Alpha (α) and Beta (β) frequency bands across several brain regions [19]. Studies focusing on regional brain activity further suggest that this is associated with altered normal patterns of hemispheric function [20]. In particular, patients with left hemisphere lesions show more symptom exacerbation, which is associated with reduced glucose metabolism in this region [21]. Research based on alpha power asymmetry has found that relatively less activity in the left frontal lobe compared to the right frontal lobe may represent the brain activity in depressed patients (since alpha power is inversely proportional to the level of activity it reflects) [22]. This asymmetry is associated with approach and avoidance motivation and has shown good internal consistency and moderate stability over time [23]. Reductions in frontal alpha power and alpha asymmetry power, measured after pharmacological treatment in depressed patients have been shown to predict treatment response [24]. In addition, depression is associated not only with local brain deficits but also with abnormal functional interactions between brain regions [25]. Some studies have found lower delta (δ) and alpha frequency bands for left and right brain connectivity in depressed patients compared to healthy individuals [26]. However, EEG signals are often influenced by brain development [27], contributing to the heterogeneity of results. Studies involving individuals with depressive symptoms have included samples of infants and adolescents [28, 29]. Among college students, a study found that those with moderate to severe depressive symptoms exhibited elevated alpha band power, which was associated with reduced cognitive abilities, decreased emotional stability, and diminished brain activity [16]. Considering the potential influence of age and behavioral patterns, coupled with the current lack of exploration into borderline depressive symptoms—a potentially critical node—we employed resting-state EEG technology to investigate whether altered brain activity exists in college students with mild depressive symptoms and the underlying neural mechanisms.

There are dynamic links between behaviour and brain activity, which underpins the search for links between behavioural patterns and associated changes in the brain [30]. For example, the decision to participate in exercise or remain sedentary is influenced in part by motivational and emotional systems, and resting EEG may have mediating effects [31]. A number of studies have linked power asymmetries with motivation for approach and avoidance, emotional experience of doing exercise [23, 32] and underlying neural activity related to depression [33]. Similar findings have been reported in studies exploring the association between meditation and brain activity. Frontal alpha power asymmetry, in particular, is expected to be a biomarker of sedentary behaviour [31]. Sleep deprivation, used as a method to reverse-explore changes in sleep behaviour, is commonly employed to better elucidate the link between sleep and depression [34]. After active changes to sleep patterns, subjects show a decrease in positive affective states and an increase in negative emotions, including stress, anxiety, and depression [35]. In addition, some studies have combined EEG indicators of wakefulness and sleep, which provides insights into the treatment of depressed patients [36]. Understanding the association between behavioural patterns and EEG indicators can provide important information for identifying possible intervention mechanisms [37]. However, there are few studies on the association between behavioural patterns and EEG signals in college students with mild depressive symptoms. The association between their behavioural patterns and resting EEG-specific indicators has not yet been validated.

Based on the analyses of previous studies, this study aims to understand the physical activity, sedentary time, sleep, and resting EEG characteristics of college students with mild depressive symptoms. Further explore the associations between behavioral patterns, resting EEG, and mild depressive symptoms. By doing so, it is hoped that this study will expand the cross-sectional evidence for future research on depression in college students.

## Methods

### Sample size Estimation

This study used the G*Power 3.1.9.4 tool for sample size estimation. Power (1-β err prob) was set as 0.80, αerr prob 0.05, and the statistical test was the difference between two independent means (two groups) with a two-sided test. The parameters were set as follows: (1) Physical activity: The variable being calculated is the total physical activity score in the IPAQ-SF. Based on prior research [38], we selected the non-depressed group with the mean of 1981.5 ± 612.72 and the depressed group with the mean of 1300 ± 847.43. The calculated effect size d was 0.922. The results showed that the required sample size was at least 20 participants per group. (2) Sedentary Time: The variable being calculated is sedentary time. Based on prior research and using a conservative data estimation method (where the original data presented sedentary time as median (interquartile range)) [39, 40], we calculated an effect size d of 0.75 between the non-depressed group (360 (180–500)) and the depressed group (480 (300–615)). The results showed that the required sample size was at least 29 participants per group. (3) Sleep: The variable being calculated is the PSQI total score. Based on previous research [41], we selected the non-depressed group with the mean of 5.19 ± 2.79 and the depressed group with the mean of 7.72 ± 3.01. The effect size d we calculated was 0.872. The results showed that the required sample size was at least 22 participants per group. (4) Resting EEG: The variable for the calculation is the power of the resting EEG. Based on prior literature [42], we selected frontal alpha asymmetry (FAA) as the computational indicator. The non-depressed group exhibited an FAA of 0.4 ± 0.5, while the depressed group showed an FAA of 0.1 ± 0.5. We calculated the effect size d to be 0.958. The results showed that the required sample size was at least 24 participants per group.

Based on the above analyses and considering the possibility of attrition during testing, we increased the planned sample size by at least 20%. This resulted in a minimum planned recruitment of 35 participants per group.

### Subject screening and recruitment process

The study adhered to the Declaration of Helsinki and was approved by Ethics Committee of the Shanghai University of Sport. Ethics number: 102772021RT004. All study subjects signed an informed consent form.

Inclusion Criteria: (1) college students aged 18 to 22 years who are not majoring in physical education. (2) Preliminary screening of target populations: ① MDS Group: college students exhibiting mild depressive symptoms (SDS cutoff score for mild depressive symptoms: 53–62 points). ② ND group: university without depressive symptoms (SDS cutoff score for non-depressive symptoms: <53 points). (3) Health status: Self-reported absence of psychiatric disorders, chronic illnesses, or history of head trauma. (4) Testing requirements: Right-handed. Normal vision or corrected to normal. No color blindness.

Exclusion criteria: (1) Assessed as having clinical depression based on an interview conducted by a physician at the university mental health center using CCMD-3 diagnostic criteria. (2) Currently taking any psychiatric medication (e.g., antidepressants, anxiolytics, hypnotics, etc.). (3) Currently suffering from any acute illness that may affect brain function (e.g., influenza, migraine) or physical capacity (e.g., musculoskeletal injury). (4) Individuals unable to complete the EEG examination for any reason.

The researchers conducted screening by posting recruitment posters at Donghua University in Shanghai and recruiting university participants before public sports elective classes. We excluded two types of invalid questionnaires: (1)Mechanical Responding: Providing identical responses across a substantial number of consecutive items༈e.g., 11,111, 22,222༉. Pattern: responding with a fixed, repeating pattern independent of item content (e.g., alternating ‘1,2,3,4,5’ or ‘3,4,3,4,3,4’). ༈2༉Inconsistent Responding: Refers to consistently affirmative responses to items that are semantically opposite or logically mutually exclusive༈e.g., reporting both “sleep duration > 7 hours” and “severe sleep insufficiency”༉。 A total of 979 valid questionnaires were ultimately collected.

College students who met the inclusion and volunteered to participate in the follow-up survey were invited for interview via telephone. Students’ past psychiatric history was self-reported in the questionnaire. To minimize the inclusion of clinically depressed individuals, we arranged for physicians with psychiatric qualifications to conduct secondary screening within the school setting. Physicians from the school’s mental health centre conducted the interviews according to the Chinese Classification and Diagnostic Criteria for Mental Disorders (CCMD-3) to exclude students with clinical depression [43]. Diagnostic Criteria for CCMD-3: Depressed mood is the primary feature, and at least 4 of the following 9 symptoms must be present: (1) Loss of interest: Loss of interest or pleasure in activities that were previously enjoyable (anhedonia). (2) Decreased energy: Marked decrease in energy, accompanied by persistent fatigue without apparent cause. (3) Psychomotor changes: Psychomotor retardation: slowed behavioral responses, reduced speech. Or agitation, restlessness. (4) Low self-esteem: Excessive self-doubt, feelings of inferiority, or unreasonable self-blame/guilt (may reach delusional levels). (5) Impaired association: Perceived significant decline in thinking ability, difficulty concentrating. (6) Sleep disturbance: Insomnia: Most typically characterized by early morning awakening (2 h or more earlier than usual). Or hypersomnia (relatively uncommon). (7) Appetite/weight changes: Markedly diminished appetite with significant weight loss (not due to dieting). Or markedly diminished appetite with significant weight loss (not due to dieting). (8) Decreased libido. (9) Recurrent thoughts of death, or suicidal ideation, attempts, and behaviors.

**Fig. 1:**
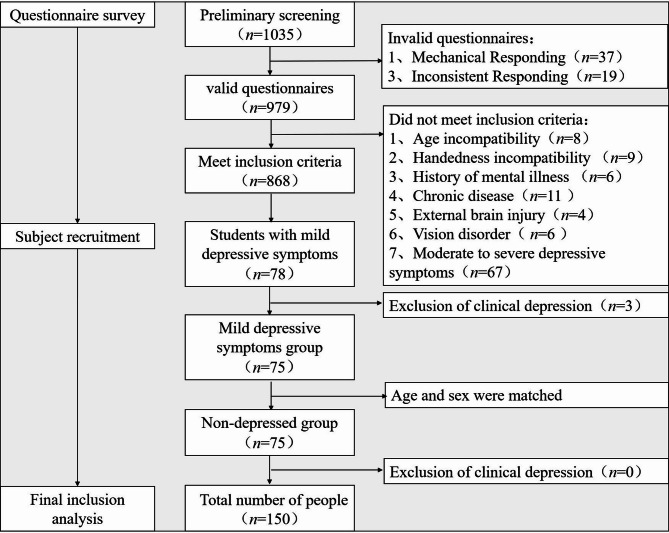
Recruitment Process

According to the age and gender of the symptomatic group, college students who were in a normal state and volunteered to participate in the follow-up survey were recruited. The screening and recruitment process is shown in Fig. 1.

### Test process

**Fig. 2:**
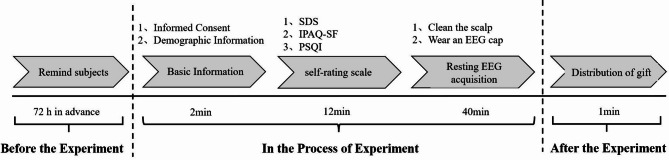
Test process

The test procedure is shown in Fig. 2. Seventy-five college students with mild depressive symptoms(MDS) and seventy-five non-depressed symptoms (ND) college students participated in the study, respectively. The researcher informed the subjects 72 h in advance. Subjects were instructed to avoid strenuous physical activities for 24 h before the test. Behaviours involving caffeine and alcohol were prohibited. Subjects were required to wash their scalps upon arrival at the testing site. The tests were conducted at the school’s Mental Health Promotion Centre. The environment was soundproof and well-ventilated, with appropriate lighting and maintained at a constant temperature of 25℃. The four EEG devices were all of the same brand and model (NCERP-190012). The tests were conducted from 10th April 2024 to 28th April 2024. The test hours were from 8:30 to 11:30 a.m., and from 14:30 to 17:30 p.m. The time spent by the two groups of subjects was divided equally between the morning and the afternoon.

### Measurement tools

#### Depressive symptoms

Depressive symptoms were assessed on the Self-Rating Depression Scale (SDS) [44], which consists of 20 questions, each question rated on a scale of 1 (none or very rarely) to 4 (overwhelmingly or all of the time.) Ten questions were stated in negative terms, while the remaining 10 were stated in positive terms and were reverse-scored. A standardised total score was used to reflect the participant’s level of depression. The statistical index of the SDS was the total score, i.e., the individual scores of the 20 items are summed to obtain the initial crude score. The standardised score is then calculated by multiplying the initial crude score by the integer part of 1.25. Higher scores indicate more severe depressive symptoms. According to the norms assessed by the Chinese Collaborative Group on Depression [45], a standardised score of < 53 suggested non-depressed symptoms, 53–62 mild depressive symptoms, 63–72 moderate depressive symptoms, and ≥ 73 severe depressive symptoms. The SDS scores of college students with mild depressive symptoms recruited for this study fell within the following range: 53–62. The scale has been translated into several languages, and studies in China have confirmed that the Chinese version has good reliability among college students. In this study, the Cronbach α coefficient was 0.89, indicating high internal consistency.

#### Physical activity and sedentary time

Physical activity and sedentary time were assessed on the International Physical Activity Questionnaire Short Form (IPAQ-SF), a valid tool that measures physical activity and sedentary time [46, 47]. The scale consists of 7 questions, with the first six questions focused on physical activity and the seventh question on sedentary time. Physical activity levels were categorised into three levels: low physical activity (LPA), moderate physical activity (MPA) and vigorous physical activity (VPA) based on the IPAQ level classification criteria. The classification was determined using metabolic equivalent (MET) values of 3.3, 4.0, and 8.0, respectively. The total physical activity level for a certain intensity (MET-min/w) is calculated by multiplying the MET value corresponding to the physical activity by the frequency per week (days) and the duration per day (minutes). The sum of the three intensity levels of physical activity yields the total physical activity level. The grouping of each physical activity level is determined by specific criteria outlined by the scale. (1) Vigorous physical activity meets any one of the following two criteria: ① All types of high-intensity physical activity ≥ 3 d, and the weekly total physical activity level ≥ 1500 MET-miw; ② Total 3 types of physical activity ≥ 7 d, and the weekly total physical activity level ≥ 3000 MET-min/w. (2) Moderate physical activity meets any 1 of the following 3 criteria: ① All types of high-intensity physical activity for at least 20 min per day for a total of ≥ 3 d; ② All types of moderate-intensity and/or walking-type activities for at least 30 min per day for a total of ≥ 5 d; ③ All 3 types of physical activity for a total of ≥ 5 d, with an overall weekly level of force activity of ≥ 600 MET-miw. (3) Low physical activity meets any of the following 2 criteria: ① No activity reported; ② The activity reported but not yet available or insufficient to meet the criteria for the medium and high groups. Total physical activity (Total PA) = VPA + MPA + LPA. Those that meet MPA or VPA standards are classified as MVPA. Sedentary behavior is determined by the self-reported sedentary time recorded on the scale. Sedentary time is quantified as a whole number. In this case, a sedentary time of ≥ 8 h/d was considered sedentary behaviour.

#### Sleep

Sleep was assessed using the Pittsburgh Sleep Quality Index (PSQI) [48], which comprises 18 question items organized into 7 sub-dimensions: ① Subjective sleep quality. ②Sleep latency. ③Total sleep duration. ④ Sleep efficiency. ⑤ Sleep disturbances. ⑥ Hypnotic medication. ⑦ Daytime dysfunction. Responses to the questions were scored from 0 to 3 points, with the total score ranging from 0 to 21 points. The cumulative score for each sub-dimension contributes to the total PSQI score. Higher the scores, poorer the sleep quality. PSQI total score ≥ 7 indicated poor sleep quality. To more intuitively translate multidimensional scores into practical screening indicators and provide intervention strategy, we attempted to adopt a binary classification approach for this study [49].

#### Resting EEG

Resting EEG signals were collected using four 16-lead EEG evoked potential meters (NCERP-190012) manufactured by Shanghai NCC Electrophysiology Co. A trained researcher applied electrode caps to the subjects and adjusted them until the scalp resistance was reduced to less than 5 kΩ. Before the test, the subjects were informed to assume a comfortable position and remain awake. A 3-minute closed-eye resting EEG was then recorded. The sampling frequency of the instrument was 256 Hz, and the electrode configuration followed the 10/20 system of electrode placement prescribed by the International Society of Electroencephalography. This system designated specific brain regions, such as the left and right prefrontal areas (Fp1, Fp2), frontal areas (F3, F4), central areas (C3, C4), parietal areas (P3, P4), fronto-temporal areas (F7, F8), occipital areas (O1, O2), mesial temporal areas (T3, T4), and posterior temporal areas (T5, T6). Reference electrodes were positioned in the bilateral earlobes (A1 and A2).

The data were analysed using EEGLAB, an open-source toolbox in the MATLAB environment, for pre-processing [16]. The interference from the industrial frequency 50 Hz AC was eliminated by concave filtering. High-pass filtering was set at 0.3 Hz, and low-pass filtering at 30 Hz. Portions of the data contaminated by eye movements, EMG or other artefacts were corrected using the independent component analysis (ICA) algorithm. Subsequently, based on frequency magnitude, the data were divided into frequency bands: δ-band (1–4 Hz), θ-band (4–8 Hz), α1-band (8–10 Hz), α2-band (10–13 Hz), β1-band (13–20 Hz), β2-band (20–30 Hz) [50, 51]. The frequency band division thresholds relate to the higher frequency bands. Spherical interpolation was applied to channels exhibiting excessive noise, drift, or poor connections. On average, 1.3 ± 0.8 channels were interpolated per subject. Eye and muscle artifacts were corrected using the ICA and IC Label toolbox. Subsequently, continuous data were segmented into 2-second trials. Trials with amplitudes exceeding ± 100 µV were automatically rejected. After pre-processing, an average of 84.2 ± 9.5% of trial data per subject were retained. Using the Fourier transform in the software, the average power for each frequency band was calculated. Data from all 150 participants were available for analysis after pre-processing.

### Statistical analysis

All data processing and graphs for this study were conducted using SPSS 29.0 and GraphPad Prism 8.0. Count data were presented as n (%) and inter-group comparison used the *χ*^*2*^ test. Measurement data that conformed to normal distribution were described as M ± SD, and inter-group comparisons were performed using the independent samples t-test. Data that did not conform to normal distribution were described as median (P25, P75), and group comparisons were made using the Mann-Whitney U nonparametric test. We applied Benjamini-Hochberg’s false discovery rate (FDR) for multiple comparison correction across 16 brain regions. The steps were: ① Sorting: sort p-values from the smallest to the largest across different brain regions. ② Ranking: assign each p-value a rank (Rank i), where the smallest p-value receives rank 1, the second smallest rank 2, and so on. ③ Calculate threshold: For each p-value, compute its FDR threshold (i _(rank)_/n _(16)_) × α _(0.05)_). ④ Identify significant p-values (p-value ≤ threshold). FDR is a well-established and widely recommended method in EEG and neuroimaging literature for handling multiple comparisons. FDR controls the expected proportion of false positives among the rejected hypotheses, which is suitable for exploratory neurophysiological studies like ours where the effects are interconnected [52]. Results with FDR-corrected p-values < 0.05 were considered statistically significant. Uncorrected p-values < 0.05 (nominally significant findings) were reported as showing a trend toward significance. Logistic regression analysis was utilized to establish associations between the variables and the occurrence of MDS, with Odds Ratio (OR) indicating the relationship for categorical variables. To assess multicollinearity among the independent variables, the variance inflation factor (VIF) was calculated, and all variables demonstrated VIF values well below 5, indicating the absence of severe multicollinearity. Correlation tests were performed using Spearman’s way test. All statistical inferences adopted method of two-sided test with a significance level (α) set at 0.05. *p* > 0.05 indicated nonsignificance, and *p* < 0.05, *p* < 0.01, and *p* < 0.001 were denoted by “*”, “**”, and “***, respectively, all of which represent statistical significance.

## Results

### Physical activity, sedentary time, sleep, and resting EEG characteristics of college students with mild depressive symptoms

As shown in Table 1, there was no statistically significant difference between the two groups in terms of age (*p* = 0.832) and gender (*p* = 0.870). However, the difference between the SDS scores of the two groups (*p* < 0.001) was statistically significant.

**Table 1:** Characteristics of the research subjects

Variable		Total Sample (*n* = 150)	Mild Depressive Symptoms(MDS) group (*n* = 75)	Non-Depressed Symptoms(ND) group (*n* = 75)	t/c^2^/Z	*p*
Demographic characteristics						
Age (years)		18.96 ± 0.767	18.95 ± 0.787	18.97 ± 0.753	−0.212	0.832
genders	male(%)	69(46)	35(46.67)	34(45.33)	0.027	0.870
	female(%)	81(54)	40(53.33)	41(54.67)		
Depressive symptoms						
SDS (score)		47.80 ± 11.129	57.56 ± 3.350	38.03 ± 6.699	22.581	<0.001^***^
Physical activity						
Vigorous physical activity (VPA) (MET-min/w)		361.81 ± 590.499	232.53 ± 483.263	491.09 ± 659.294	−2.793	0.007^**^
Moderate physical activity (MPA) (MET-min/w)		456.03 ± 445.558	332.59 ± 381.104	579.47 ± 472.863	−3.520	<0.001^***^
Low physical activity (LPA) (MET-min/w)		780.67 ± 344.604	737.36 ± 310.212	823.97 ± 372.928	−1.546	0.124
Total PA (MET-min/w)		1580.98 ± 930.806	1302.48 ± 847.596	1859.48 ± 932.131	−3.829	<0.001^***^
Sedentary time						
Sedentary time(h/day)		7.17 ± 2.141	7.99 ± 2.153	6.35 ± 1.797	5.065	<0.001^***^
Sleep quality						
Subjective sleep quality(score)		0.81 ± 0.748	1.11 ± 0.781	0.51 ± 0.578	5.347	<0.001^***^
sleep latency(score)		0.84 ± 0.883	1.19 ± 0.982	0.49 ± 0.601	5.214	<0.001^***^
sleep duration(score)		0.81 ± 0.754	1.13 ± 0.777	0.49 ± 0.578	5.723	<0.001^***^
Sleep efficiency(score)		0.44 ± 0.831	0.64 ± 0.968	0.24 ± 0.612	3.025	0.003^**^
Sleep disturbances(score)		0.82 ± 0.544	0.96 ± 0.556	0.68 ± 0.498	3.250	0.001^**^
Hypnotic drug use(score)		0.09 ± 0.407	0.19 ± 0.562	0	-	-
Daytime dysfunction(score)		1.25 ± 1.044	1.77 ± 0.909	0.73 ± 0.905	7.020	<0.001^***^
Total PSQI(score)		5.07 ± 3.533	6.99 ± 3.423	3.15 ± 2.437	7.915	<0.001^***^
Resting-state EEG						
Delta (δ) band (µv^2^)		1.43(1.19, 1.66)	1.46(1.25,1.64)	1.39(1.14,1.66)	0.614	0.504
Theta (θ) band (µv^2^)		9.55(7.94,11.08)	9.65(8.23,11.13)	9.14(7.81,10.95)	−0.739	0.460
Alpha1 (α1) band (µv^2^)		7.48(5.48,10.99)	7.46(5.20,10.83)	7.77(5.59,11.60)	0.184	0.854
Alpha2 (α2) band (µv^2^)		14.28(11.15, 19.02)	16.03(11.04,19.55)	13.34(11.44,17.96)	−1.120	0.263
Beta1 (β1) band (µv^2^)		8.28(7.09,9.77)	8.41(7.20,9.96)	8.04(6.88,9.58)	−0.735	0.462
Beta2 (β2) band (µv^2^)		12.59(10.68,14.56)	12.30(10.61,14.60)	12.62(10.81,13.86)	−0.098	0.922

Total PA = VPA + MPA + LPA. (2) The cumulative score for each sub-dimension equals the total PSQI score

**Fig. 3:**
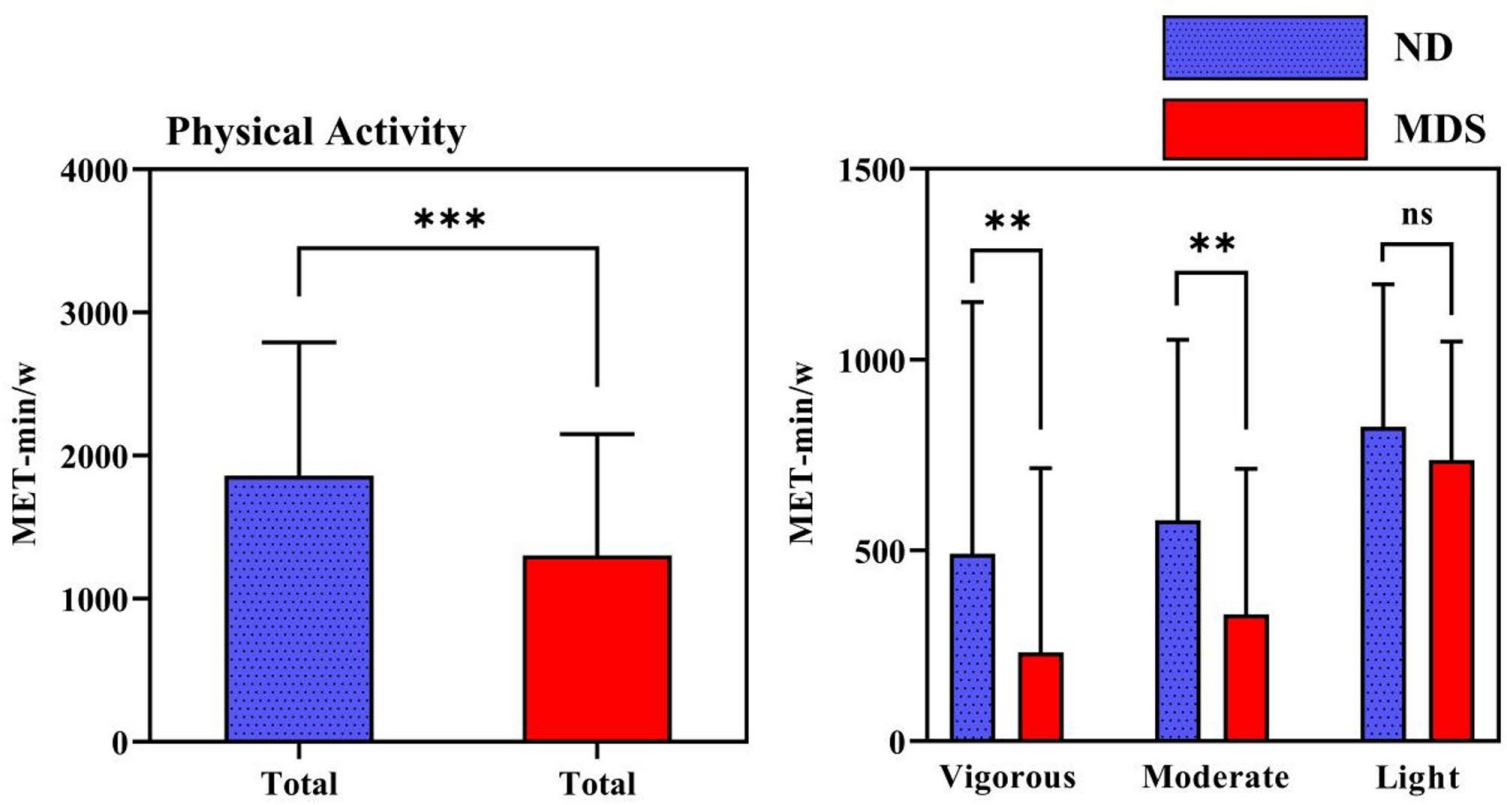
Comparison of physical activity between the two groups

As can be seen in Fig. 3; Tables 1 and 2, the VPA in the MDS group was lower than that of the ND group (t=−2.793, *p* = 0.007). The MPA in the MDS group was lower than that of the ND group (t=−3.520, *p* < 0.001). However, the difference in the LPA between the MDS group and the ND group was not statistically significant (t=−1.546, *p* = 0.124). The total PA in the MDS group was lower than that of the ND group (t=−3.829, *p* < 0.001). The MPA and VPA in the MDS group were lower than those in the ND group. MPA referred to those that meet MPA or VPA standards are classified [53]. Analysis of MVPA behaviours between the two groups revealed that 56% of MDS college students exhibited MVPA behaviours, compared to 84% of ND college students, indicating lower MVPA behaviours among MDS college students (*c*^*2*^ = 20.953, *p* < 0.001).

**Table 2:** Behavioral patterns of the research subjects

variable		MDS group (*n* = 75)	ND group (*n* = 75)	c^2^	*p*
Moderate-to-Vigorous Physical Activity (MVPA)	Yes(%)	42(56)	63(84)	20.953	< 0.001^***^
Sedentary Behavior (SB)	yes(%)	46(61)	15(20)	26.552	< 0.001^***^
Poor sleep quality (PSQ)	yes(%)	32(43)	3(4)	31.342	< 0.001^***^

**Fig. 4:**
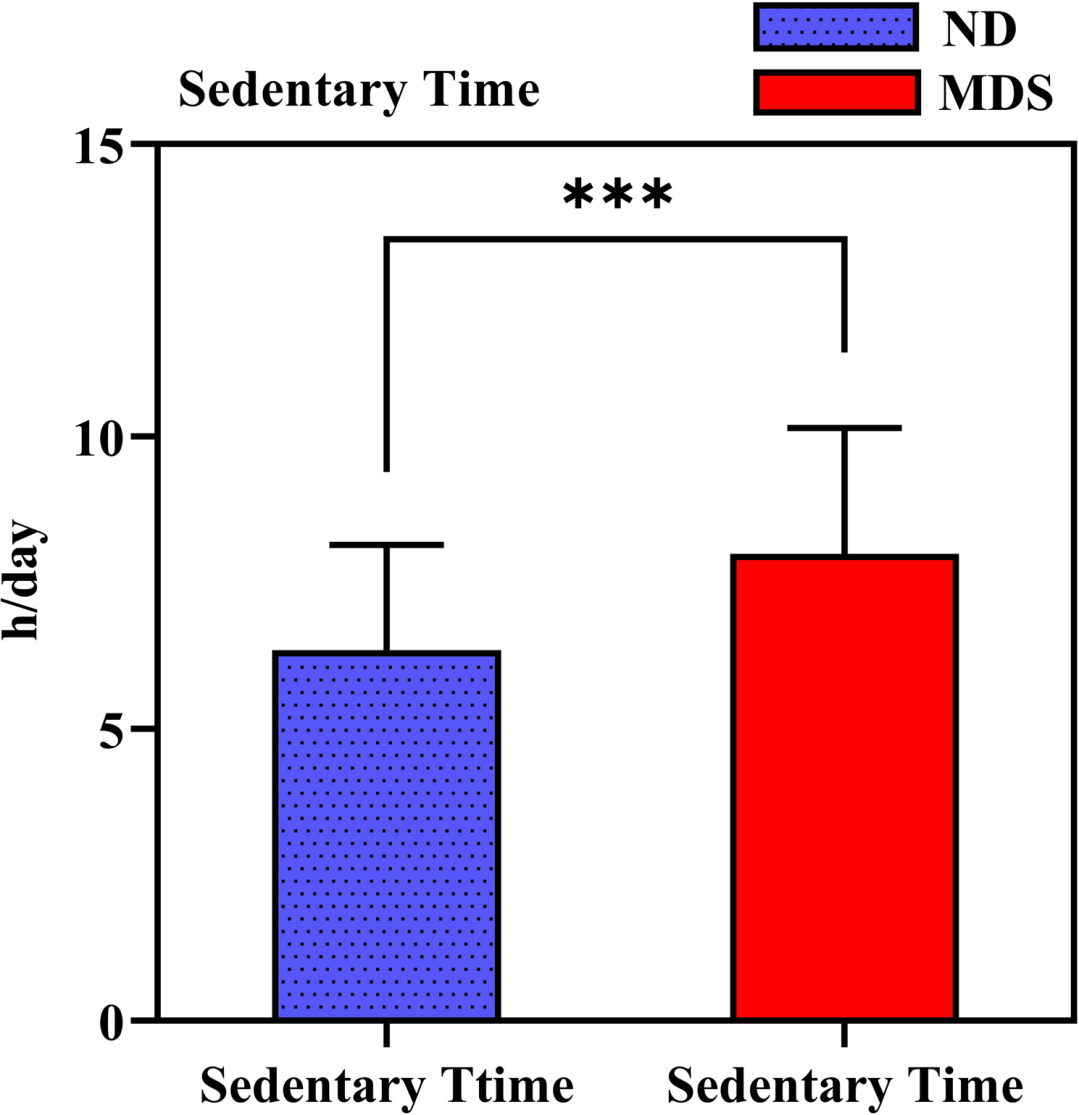
Comparison of sedentary time between the two groups

As seen in Fig. 4; Tables 1 and 2, sedentary time was higher in the MDS group than in the ND group (t = 5.065, *p* < 0.001). SB analysis revealed that 61% of the MDS college students exhibited SB, while 20% of the ND college students did so, indicating higher SB among MDS students (*c*^*2*^= 26.552, *p* < 0.001).

**Fig. 5:**
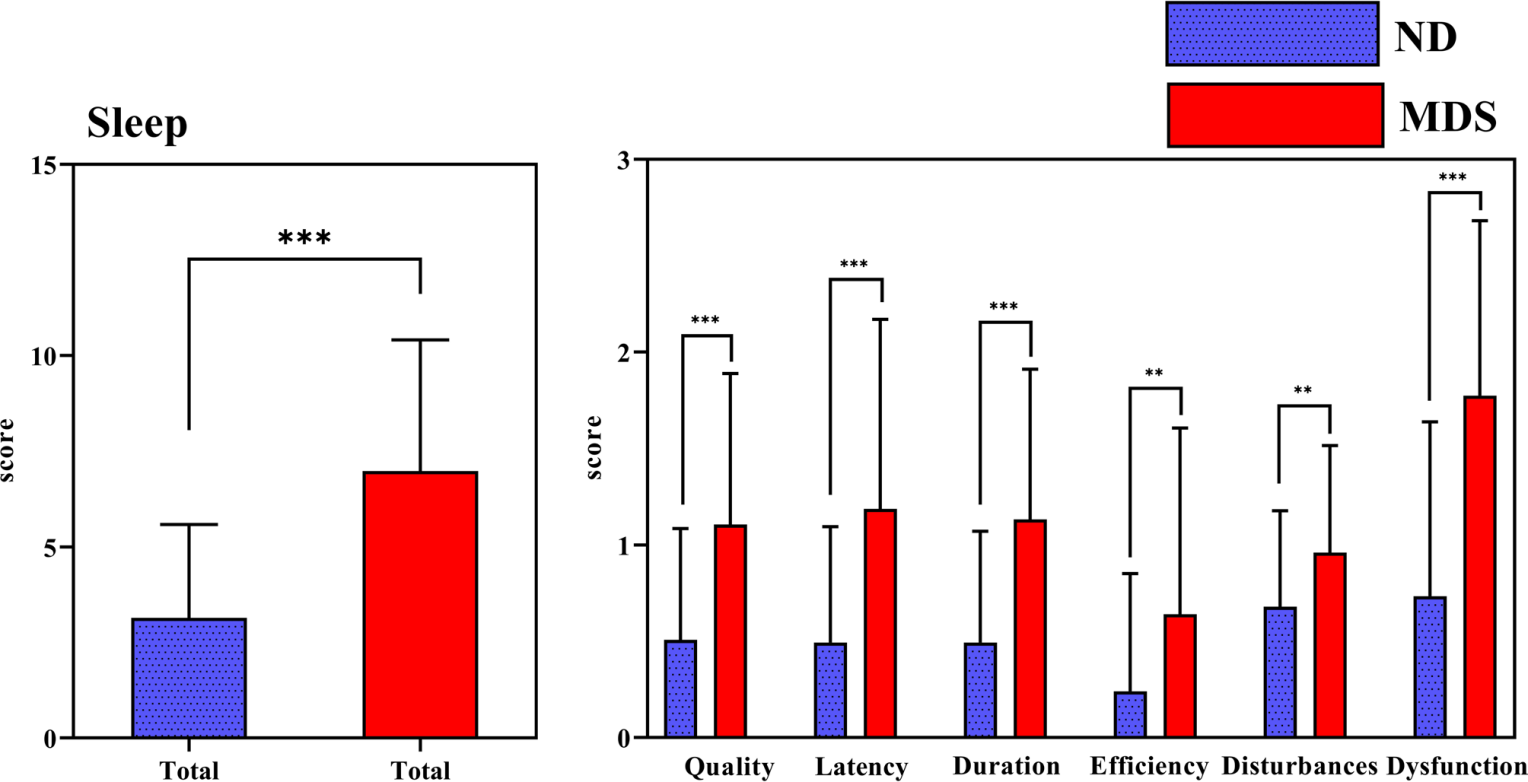
Comparison of PSQI scores between the two groups

As seen from Fig. 5; Tables 1 and 2, the total PSQI score of the MDS group was higher than that of the ND group (t = 7.915, *p* < 0.001). Across PSQI dimensions, the subjective sleep quality score of the MDS group was higher than that of the ND group (t = 5.347, *p* < 0.001). Additionally, the MDS group exhibited higher scores for sleep latency (t = 5.214, *p* < 0.001), sleep duration (t = 5.723, *p* < 0.001), sleep efficiency (t = 3.025, *p* = 0.003), sleep disturbances (t = 3.250, *p* = 0.001), and daytime dysfunction (t = 7.020, *p* < 0.001) compared to the ND group. The ND group had a score of 0 in the hypnotic drug use score, so it was not presented in the graph. PSQ behaviours were analysed between the two groups. PSQ was present in 43% of the MDS college students compared to 4% of the ND college students, indicating higher PSQ behaviours among MDS college students (*c*^*2*^ = 31.342, *p* < 0.001).

**Fig. 6:**
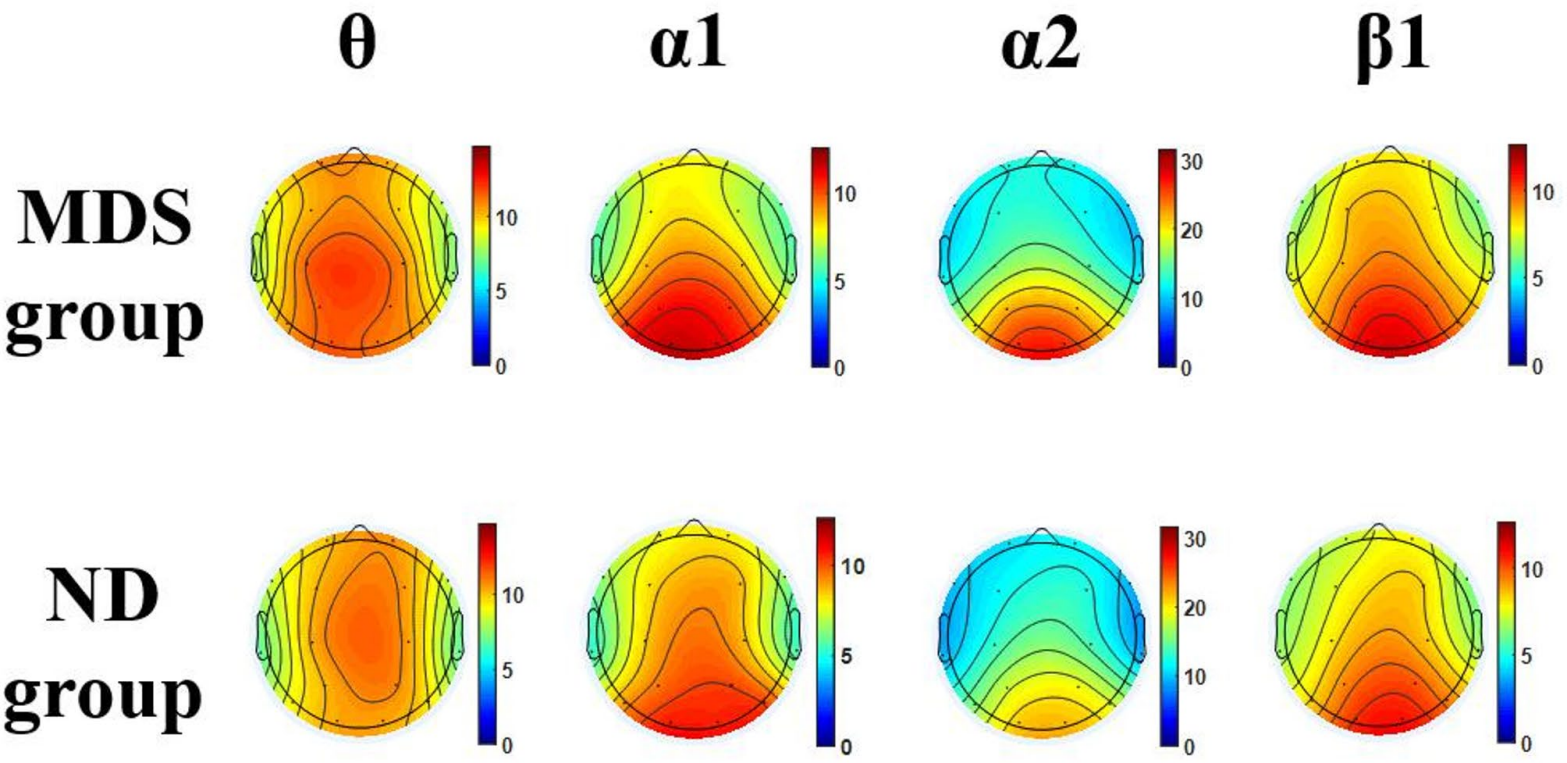
Comparison of brain topography between the two groups

As seen from Table 3; Fig. 6, the theta band power of C3 brain region (z = 2.331, *p* = 0.020), P3 brain region (z = 2.556, *p* = 0.011), and T3 brain region (z = 2.685, *p* = 0.007) was higher in the MDS group compared to the ND group. Conversely, The α1 band power of F4 brain region (z=−1.981, *p* = 0.048) was lower in the MDS group than in the ND group. the α2 band power of Fp1 brain region (z = 2.314, *p* = 0.021), P3 brain region (z = 2.532, *p* = 0.011), O1 brain region (z = 2.265, *p* = 0.024), T3 region (z = 3.109, *p* = 0.002), and T5 brain region (z = 2.793, *p* = 0.005) had higher α2 band power than the ND group. The β1 band power in the Fp1 (z = 3.077, *p* = 0.002) and F3 (z = 2.278, *p* = 0.023) brain regions was higher in the MDS group than in the ND group.

**Table 3:** Comparison of band power at different brain region locations between the two Groups(Indicators with statistical Differences)

Variable		MDS group(*n* = 75)	ND group (*n* = 75)	Z	*P*	Rank (i)	Threshold (i/16)×0.05	Whether significant after correction (*p* ≤ Threshold)
(µv^2^)		M(*P*_25_, *P*_75_)	M(*P*_25_, *P*_75_)					
Theta (θ) band (µv^2^)	T3	7.1(5.9, 8.8)	6.2(5.4, 7.5)	2.685	0.007^**^	1	0.003125	No (0.007 >0.003125)
	P3	10.9(9.1, 12.9)	9.6(7.8, 11.7)	2.556	0.011^*^	2	0.00625	No 0.011 >0.00625
	C3	10.7(8.8, 12.7)	9.5(7.6, 12.7)	2.331	0.020^*^	3	0.009375	No 0.020 >0.009375
Alpha1 (α1) band (µv^2^)	F4	6.2(4.7, 8.8)	7.8(5.1, 11.1)	−1.981	0.048^*^	1	0.003125	No 0.048 >0.003125
Alpha2 (α2) band (µv^2^)	T3	10.5(7.9, 13.5)	8.9(6.8, 11.0)	3.109	0.002^**^	1	0.003125	YES 0.002 < 0.003125
	T5	17.8(12.1, 24.8)	13.7(10.0, 20.6)	2.793	0.005^**^	2	0.00625	YES 0.005 < 0.00625
	P3	19.7(13.5, 27.7)	16.0(11.8, 21.8)	2.532	0.011^*^	3	0.009375	No 0.011 >0.009375
	Fp1	12.2(9.0, 14.9)	10.3(8.3, 13.2)	2.314	0.021^*^	4	0.0125	No 0.021 >0.0125
	O1	24.5(15.8, 31.5)	19.4(14.2, 25.8)	2.265	0.024^*^	5	0.015625	No 0.024 >0.015625
Beta1 (β1) band (µv^2^)	Fp1	7.9(6.6, 8.6)	6.8(5.7, 8.1)	3.077	0.002^**^	1	0.003125	YES 0.002 < 0.003125
	F3	8.0(6.6, 9.6)	7.3(5.8, 9.0)	2.278	0.023^*^	2	0.00625	No 0.023 >0.00625

Due to the non-normal distribution of EEG power data and its collection across 16 brain regions, we employed the FDR method for multiple comparison correction of EEG data. Analysis after FDR correction revealed that in the α2 band, the MDS group exhibited higher power values than the ND group in the T3 region (*p*_*FDR*_ = 0.002) and the T5 region (*p*_*FDR*_ = 0.005). In the β1 band, the MDS group demonstrated higher power values than the ND group in the Fp1 region (*p*_*FDR*_ = 0.002).

### Exploratory analysis

#### Correlation between behavioral patterns and mild depressive symptoms

To more intuitively translate multidimensional scores into practical screening indicators and provide intervention strategy, A binary classification approach was used to explore the correlation between behavioral patterns and mild depressive symptoms [47, 48]. Indicators that differed between college students with MDS and ND students were selected for correlation analysis. See Table 4 for an analysis of MDS occurrence (yes = 1, no = 0) as the dependent variable, with MVPA behaviour, SB, and PSQ behaviour incoporated into the multifactorial logistic regression equation.

**Table 4:** Correlation between behavioral patterns and mild depressive symptoms(n = 150)

Independent variable	Assign value	B	SE	Wald	*P*	OR(95%CI)	VIF
MVPA (Moderate-to-Vigorous Physical Activity)	No = 1, Yes = 2	−1.395	0.518	0.518	0.007^**^	0.248 (0.090, 0.684)	1.241
SB (Sedentary Behavior)	No = 1, Yes = 2	1.276	0.422	9.166	0.002^**^	3.583 (1.568, 8.187)	1.249
PSQ (Poor sleep quality)	No = 1, Yes = 2	2.382	0.668	0.668	<0.001^***^	10.829 (2.923, 40.112)	1.180
Constant	-	−2.077	1.388	2.238	0.135	0.125 (0.008, 1.905)	-

Logistic regression models were included using the entry method(Table 4). The pooled test of model coefficients showed (*c*^*2*^ = 56.761, *p* < 0.001), with *P* < 0.05 indicating that at least one of the variables included in this fitted model had a statistically significant OR, i.e., the model was significant overall. The goodness-of-fit test for the model (Hosmer and Lemeshow Test) showed (*c*^*2*^ = 0.439, *p* = 0.932), indicating *p* > 0.05. This suggested that the information in the current data was adequately extracted, and the model exhibited good fit.

**Fig. 7:**
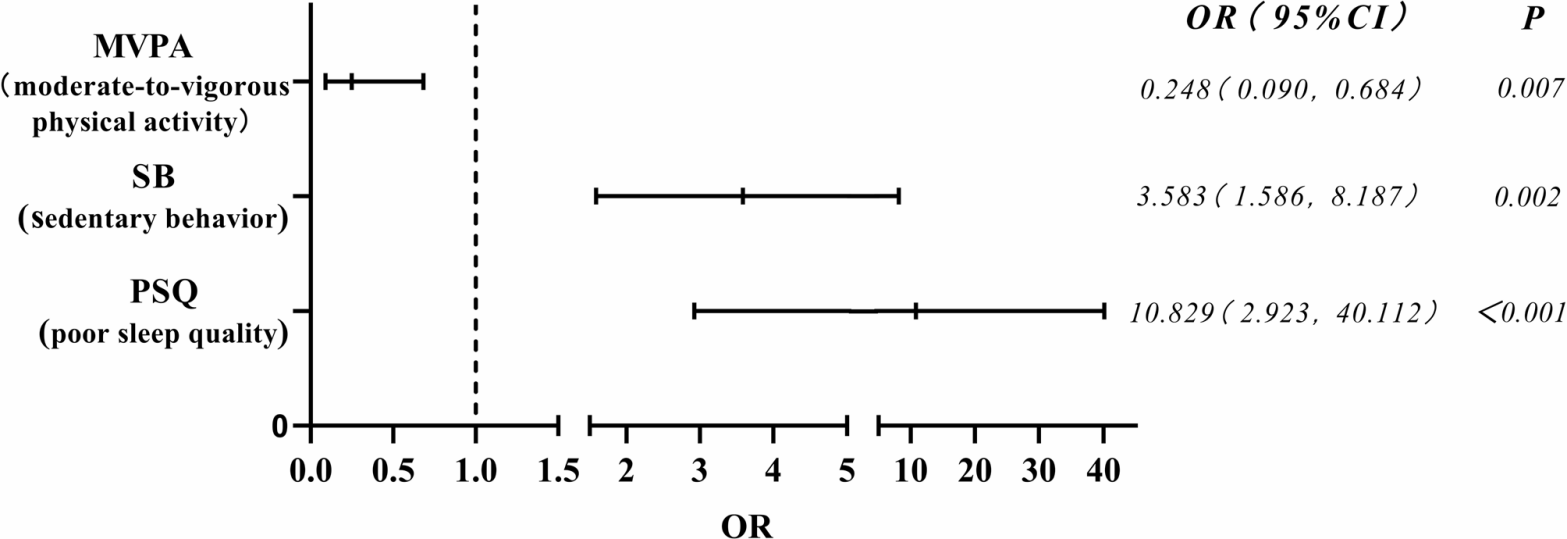
Correlation between behavioural patterns and mild depressive symptoms

Table 4; Fig. 7 illustrates that with MVPA, SB, and PSQ included in the regression model, there was no multicollinearity between the indicators (all VIF < 5). SB (*p* = 0.002) and PSQ (*p* < 0.001) behaviors may be positively correlated with mild depressive symptoms, while MVPA (*p* = 0.007) behavior may be negatively correlated with mild depressive symptoms.

#### Correlation between resting EEG and mild depressive symptoms

To explore the EEG patterns of college students with mild depressive symptoms at a deeper level, this study is exploratory in nature to some extent, aiming to provide potential evidence for future research. As seen in Table 5, the presence of MDS (coded as 2 for yes and 1 for no) served as the dependent variable. We selected two sets of brain electrical indicators with statistically significant differences between groups (after correction) as independent variables. Logistic regression models were included using the entry method. The pooled test of model coefficients showed (*c*^*2*^ = 13.413, *p* = 0.004), with *P* < 0.05 indicating that at least one of the variables included in this fitted model had a statistically significant OR, i.e., the model was significant overall. The goodness-of-fit test for the model (Hosmer and Lemeshow Test) showed (*c*^*2*^ = 4.116, *p* = 0.846), indicating *p* > 0.05. This suggested that the information in the current data was adequately extracted, and the model exhibited good fit. Table 5 illustrates that with T3α2, T5α2, and Fp1β1 included in the regression model, there was no multicollinearity between the indicators (all VIF < 5).

**Table 5:** Inclusion of variables in the resting EEG regression Equation(*n* = 150)

Variable	B	SE	Wald	*P*	OR(95%CI)	VIF
T3α2	0.095	0.059	2.577	0.108	1.100(0.979, 1.235)	1.576
T5α2	0.020	0.028	0.526	0.468	1.020(0.966, 1.078)	1.578
Fp1β1	0.106	0.102	1.084	0.298	1.112(0.911, 1.356)	1.212
Constant	−2.109	0.752	7.866	0.005	0.121(0.028, 0.530)	-

**Fig. 8:**
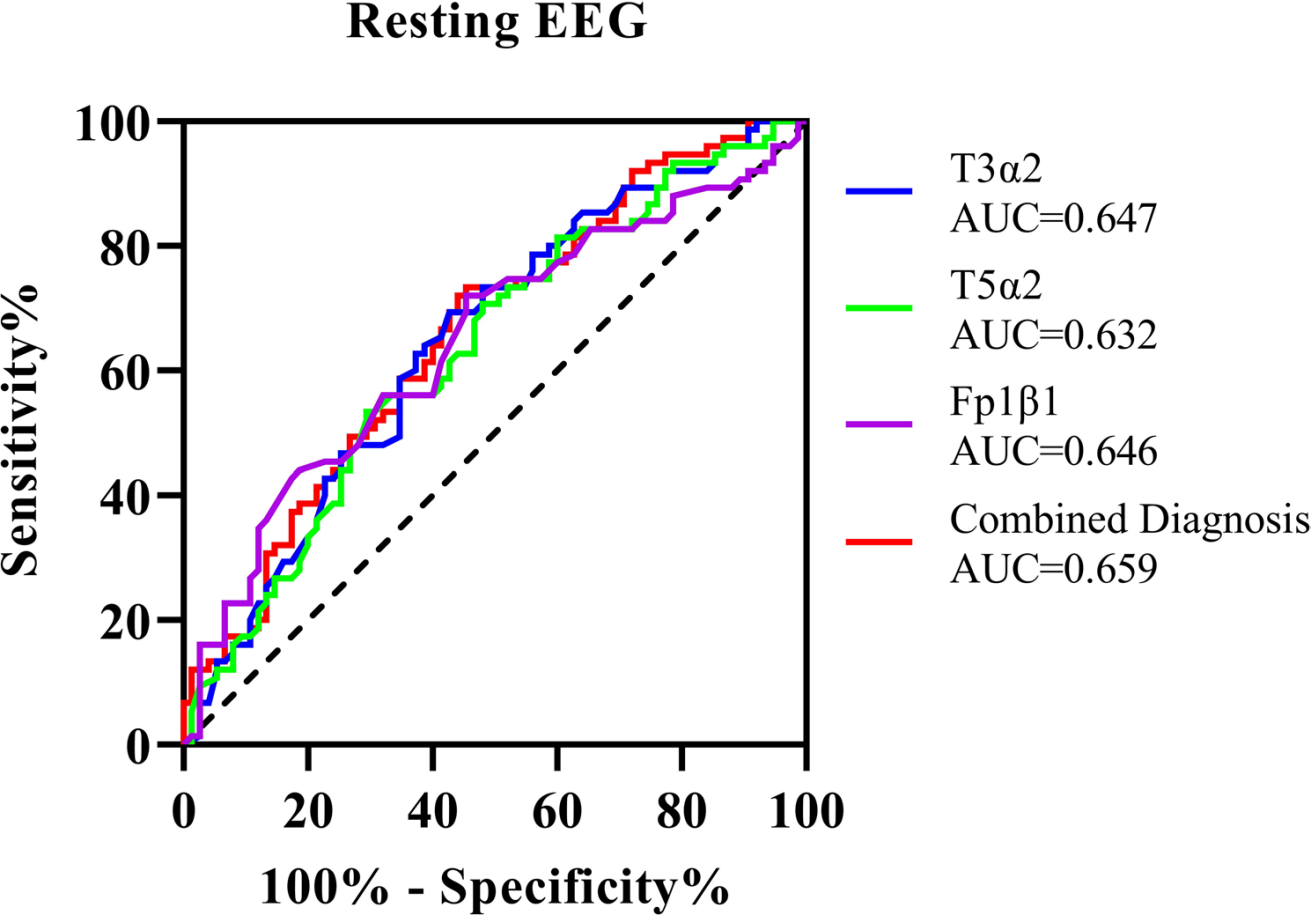
ROC curve for identifying mild depression symptoms in college students using selected resting-state EEG indicators

As shown in Table 6; Fig. 8, we attempted to use the α2 band power from the T3 region, the α2 band power from the T5 region, and the β1 band power from the Fp1 region for combined identification of mild depressive symptoms. The area under the curve (AUC) was 0.659 (95% CI: 0.572 to 0.745, *p* = 0.001), with a sensitivity of 0.560 and a specificity of 0.720.

**Table 6:** Identification of mild depression symptoms in college students using Resting-State EEG Indicators(*n* = 150)

Variable	AUC	SE	*P*	95%CI	Optimal threshold	Sensitivity	Specificity	Cut-off
joint identification	0.659	0.044	0.001^**^	0.572, 0.745	0.280	0.560	0.720	0.520
T3α2	0.647	0.045	0.002^**^	0.559, 0.735	0.267	0.573	0.693	9.800
T5α2	0.632	0.045	0.005^**^	0.543, 0.721	0.240	0.707	0.533	13.900
Fp1β1	0.646	0.045	0.002^**^	0.557, 0.734	0.267	0.547	0.720	7.700

#### Correlation Between Behavioral Patterns of College Students with Mild Depressive Symptoms and Potential Specific Resting EEG Indicators

**Fig. 9:**
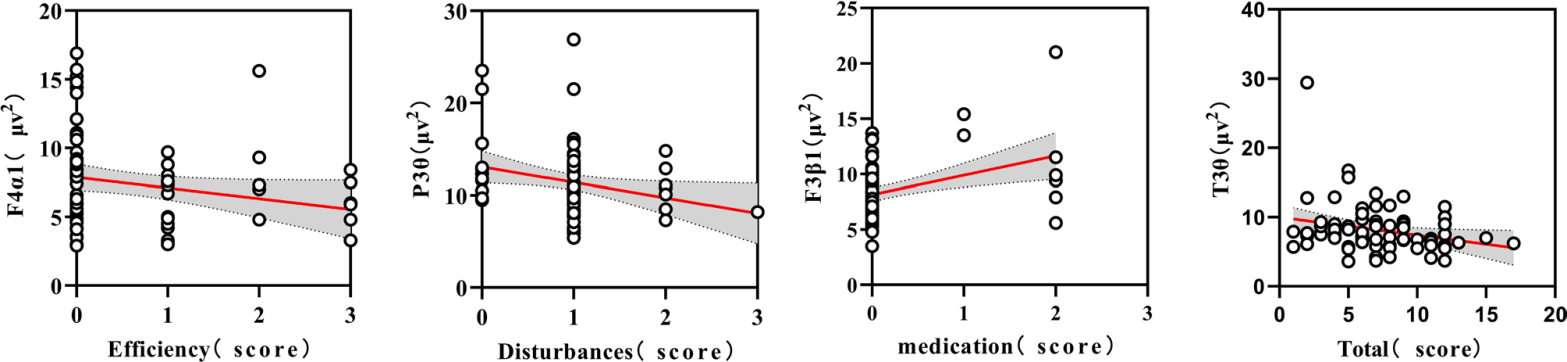
Correlation between sleep and potential specific resting EEG indicators

To avoid overlooking biologically plausible associations and conditions that worth further hypothesis-driven investigation, the potential specific resting EEG indicators selected in this section included both those showing significant differences between groups after multiple-region correction and those exhibiting significant differences prior to correction. This approach aims to capture a broader range of potential neurophysiological associations. As shown in Fig. 9, we found that in the behavioral patterns of university students with mild depressive symptoms, sleep efficiency scores showed a significant negative correlation with α1 power in the F4 region (*r* = −0.254, *p* = 0.028). Additionally, there was a negative correlation between the sleep disturbances score and the θ power of the P3 brain region (*r*=−0.231, *p* = 0.046). Furthermore, a positive correlation was identified between the hypnotic drug score and the β 1 power of the F3 brain area (*r* = 0.275, *p* = 0.017). Lastly, a negative correlation was observed between the total PSQI and θ power in the T3 brain area (*r*=−0.238, *p* = 0.040).

## Discussion

We found that college students with MDS had lower moderate-to-vigorous physical activity levels than college students with non-depressed symptoms. This is consistent with similar studies. Previous studies have also indicated lower levels of physical activity in older adults and adolescents with depressive symptoms [54, 55]. The features of reduced physical activity corresponds to the behaviours of avoidance motivation and an active negative emotional system in depressive symptom descriptions [16]. Additionally, our study indicates that college students with mild depression symptoms exhibit lower levels of moderate-to-vigorous physical activity compared to college students with non-depressed symptoms, suggesting that moderate-to-vigorous physical activity is associated with a lower risk of depressive symptoms. Physical activity has been shown to be positive against depressive symptoms in adolescents and older adults, as high levels of physical activity have significantly lower odds of developing depressive symptoms compared to those with low physical activity [56]. Importantly, there was a significant negative dose-effect relationship between physical activity and depressive symptoms, and high-intensity physical activity showed significant benefits in symptom reduction, whereas low-intensity physical activity did not [57]. A follow-up study found that while high school students often participated in regular exercise, many tended to discontinue this habit upon entering college, which dramatically increased their risk of having mental health diseases [6]. The depressed vulnerability due to the lack of moderate-to-vigorous physical activity may be related to the reward effect [58]. In this regard, social-behavioural cognitive theory suggests that the psychological processes surrounding moderate-to-vigorous physical activity, which typically require dealing with difficult tasks, such as perceived achievement and self-efficacy, contribute positively to the overall psychological climate of individuals [59]. In addition, depression-prone populations generally have insufficient secretion of monoamine neurotransmitter levels in the brain [58]. Physical activity enhances the secretion of 5-hydroxytryptamine, dopamine, and endorphins, which play a crucial role in maintaining a balance of neurotransmitter secretion [59]. In terms of exercise intensity, the American College of Sports Medicine recommends an intensity of 60%−80% of MHR to prevent and treat depression [60].

We found that college students with mild depression symptoms had higher levels of sedentary time than college students with non-depressed symptoms, which is consistent with similar studies. Previous studies have shown that children, adolescents, and perinatal women with depressive symptoms exhibit significantly higher sedentary time, featuring increased sedentary behaviours [61]. Additionally, our research found that sedentary behaviours is more prevalent among college students experiencing depressive symptoms, indicating that sedentary behaviours is associated with a higher likelihood of depressive symptoms. Previous studies have confirmed a positive correlation between screen time and the likelihood of depressive symptoms in adults, and screen time is propotional to depressive symptoms in older adults [62]. This phenomenon of higher rates of depressive symptoms triggered by sedentary behaviours can be supported by social cognitive theory [63]. The social withdrawal-isolation hypothesis suggests that chronic sedentary behaviours may lead to feelings of loneliness, which can have a negative impact on mental health [64]. In addition, sedentary behaviours have been linked to the accumulation of visceral fat, elevated inflammation in adipose tissue, and, eventually, a chronic systemic inflammatory state [65]. Elevated systemic inflammatory factors may triggerring the potential link between sedentary behaviours and the development of depressive symptoms [66]. In addition, the depression vulnerability due to sedentary behaviours is closely related to alterations in brain metabolism, particularly changes in brain-derived neurotrophic factor (BDNF) levels, which is reported to have negative correlation with sedentary behaviours in related studies [63]. Although we found that sedentary behaviours are associated with a higher likelihood of depressive symptoms, they may also be caused by mild depression symptoms, leading to a vicious cycle [67]. One of the core symptoms of depression is a loss of motivation and diminished pleasure in previously enjoyable activities, which can lead individuals to become less willing to engage in physically demanding activities and instead adopt sedentary behavioral patterns [68]. The biological mechanism between depressive symptoms and sedentary behaviours may involve bidirectional feedback pathways. For example, depressive symptoms may lead to increased sedentary time, which in turn exacerbates inflammatory processes, reduces behavioral activation, and thereby worsens depressive symptoms [69]. For future research, we recommend establishing a longitudinal cohort study model with repeated measurements. This would help clarify the temporal sequence between sedentary behavior and depressive symptoms. Additionally, incorporating interventions targeting sedentary behavior within the cohort could further deepen our understanding of the relationship between the two.

Our findings indicate that college students with mild depression symptoms had lower sleep quality than college students with non-depressed symptoms, which is consistent with similar studies. Decreased sleep quality often precedes the development of depressive symptoms and is frequently reported as the primary concern among help-seeking individuals with depressive symptoms [70]. Previous research has noted the prevalence of poor sleep qualityamong adolescents with depressive symptoms and its tendency to exacerbate with age [71]. For instance, a study found significantly longer self-reported sleep latency and reduced sleep duration among postpartum women with depressive symptoms [72]. Our findings further indicate that poor sleep quality is associated with a higher likelihood of depressive symptoms. A positive correlation between adolescent PSQI scores and their depressive symptoms has been shown in previous studies [73]. Students with poor sleep quality are approximately 3.28 times more likely to develop depressive symptoms [74]. Sleep quality is often overlooked by college students during their transition from high school, where they may have more fixed routines, to college, where they enjoy greater autonomy [75]. Biological clock is a timing mechanism to maintain various internal physiological activities, such as sleep-wake cycles and hormone regulation [76]. Polymorphisms in biological clock genes are highly correlated with mood disorders. It has been found that depressed individuals show reduced expression of core biological clock genes, such as Bmal1 and Per1-3, in different brain regions (e.g., dorsolateral prefrontal, amygdala, anterior cingulate cortex, and hippocampus) [77]. The internal-conformity model proposes that individuals sleeping out of their biological clock time may suffer a misalignment between their biological clock and the sleep-wake cycle, resulting in depressive symptoms [34]. In addition, melatonin regulates sleep, ageing, immune responses, and mood. Reduced sleep duration at night affects melatonin secretion and increases the risk of developing depression [78]. In addition, depression is associated with dysregulation of the HPA axis and abnormal glucocorticoid secretion. Biological clock genes help maintain normal bodily functioning by stabilizing monoamine neurotransmitters, HPA axis pathways and neural signalling pathways, which in turn regulate the transcription and translation of target genes [34].We found that poor sleep quality is associated with the likelihood of depressive symptoms, but depressive symptoms may also lead to worsened sleep quality. For example, a key feature of depressive symptoms is rumination—a repeated mental replaying of negative events [79]. This tendency is particularly pronounced at night, when rumination is most likely to occur. The brain remains in a state of heightened cognitive arousal due to negative thoughts, which contradicts the relaxed state required for sleep, leading to difficulty in falling asleep and fragmented sleep [80]. To address this issue, there is an urgent need for future longitudinal studies with repeated measurements to map the temporal dynamics between sleep and mood.

We observed alterations in the indicators of some brain regions in the alpha (α), and beta (β) frequency bands in college students with mild depression symptoms compared to college students with non-depressed symptoms. Additionally, we observed a significant trend of difference between the two groups in the theta (θ) frequency band. The results of this study support the conclusion that the brains of depressed individuals may exhibit abnormalities. We selected power spectrum analysis because this method is classical and well-established, which potentially facilitates expansion and comparison with existing similar literature [81]. Previous studies have linked the theta band to emotional processing, and elevated power values in the theta band indicate a state of stress or negative emotions [82]. Related studies have noted elevated theta frequency bands in central, parietal, and temporal regions in depressed individuals [83]. These abnormalities in depressive theta oscillations are associated with alterations in the limbic-cortical pathway, reflecting disrupted connections in the cingulate pathway that regulates emotion [84]. Specifically, theta power values in the anterior cingulate cortex (ACC) region significantly predict the response to antidepressant treatments, whereas theta power values in the central and parietal-lateral regions can assist in the identification of depressive disorders [85]. In addition, the alpha frequency band is a signature rhythm of the physiological state of the brain, related to cognition and memory [86]. Depressed patients normally exhibit elevated alpha frequency bands in the left hemisphere (T3, F7, O1, P3) and right hemisphere (C4) of the brain, in which the alpha 2 frequency band shows more significant differences compared with the alpha 1 frequency band [87]. Increased alpha power in brain regions is associated with hypervigilant behaviour and reduced cognitive function in depressed individuals [88]. It has been further suggested that brain-derived neurotrophic factor (BDNF) is lower in depressed individuals than in normal individuals and that EEG α power may mediate the association between depression and BDNF gene polymorphisms, thus reflecting abnormal changes in the excitatory-inhibitory pathway in the depressed population [89]. In addition, beta rhythms are associated with cortical excitability, reflecting the emotional and cognitive processes of individuals [17]. Increased beta-band power values are associated with elevated negative affect and attention deficits [90]. Increased absolute beta-band power values in clinically depressed patients are consistently found across several studies [91]. Previous studies have identified a significant positive correlation between high beta oscillations and both the number and severity of depressive relapses [92]. It is now widely accepted that changes in the human brain, including levels of neurotransmitters that play a role in neural transmission and the process of neural signal propagation, cause changes in the brain’s neurophysiological signals. In particular, depression affects brain activity in almost the entire cortex and is reorganised by abnormal EEG oscillations [93]. This results in a disturbance of distributed EEG oscillations, reflecting altered brain function in its resting state. Our research also found that the recognition model based on certain EEG indicators exhibits preliminary recognition efficacy. Currently, the scientific community is using EEG to better understand the mechanisms of depression and develop objective identification models for more accurate discrimination [94]. For instance, in a comparison between clinically depressed male patients and healthy controls, the patients showed higher absolute beta power in bilateral frontal regions, and a classification model based on absolute power and coherence obtained a recognition probability of 91.3% [95]. The alpha and theta bands were found to be good classifiers of clinically depressed patients versus normal subjects, with an 88.33% probability of identification in a survey of 90 subjects [94]. An average identification probability of 80% was obtained by selecting the absolute power of delta, theta, alpha and beta bands from 96 subjects [19]. Currently, some studies describe depression as a dysfunction of the brain’s cortical-limbic pathway network [96]. This suggests that depression may result from the dysfunctional ability of brain cells to communicate with each other, indicating the potential for objective identification and optimal therapeutic evaluation using brain-based classification algorithms.

We found that sleep problems in college students with mild depression symptoms correlate with their underlying specific resting EEG indicators. These correlations may indicate a subtle, potential link between behavioral patterns and neurophysiological functions. Brain slow-wave activity is associated with deeper, better-quality sleep involving metabolic regulatory homeostasis [97]. In contrast, physiological hyperarousal, such as increased fast-frequency activity (β-activity), often observed in depressive symptoms, may lead to difficulty in falling asleep or disturbed sleep [98]. In this regard, the endosynaptic homeostasis hypothesis suggests that reduced slow-wave activity during sleep leads to insufficient synaptic incapacitation, which in turn leads to synaptic overload upon awakening [36]. This synaptic overload can cause reduced neuronal excitability, increased synaptic failures, and lower plasticity. These neural abnormalities are also reflected in depressive symptom profiles, such as fatigue, lack of pleasure, and impaired attention [99]. The asymmetry of left and right frontal alpha power reflects individual differences in emotion regulation and has a strong association with depressive symptoms [36]. Due to the inhibitory effect of the alpha band on cortical network activity, alpha power is inversely related to the corresponding level of cortical activity [100]. For instance, relative activation in the right frontal lobe (reduced alpha power) is associated with increased negativity during sleep and sleep abnormalities, compared to those with more activation in the left frontal lobe [101].

This study has some limitations: (1) The sample for this study consisted of college students exhibiting mild depressive symptoms, not individuals clinically diagnosed with major depressive disorder. The findings should be interpreted with caution. (2)This study relied on subjects’ subjective recall of physical activity, sedentary time, and sleep, which may have recall bias. Future research should incorporate objective measurement devices, such as physical activity accelerometers and sleep EEG detectors, to enhance accuracy. Future studies should use objective equipment to verify and clarify the different cut-off points of classification after continuous segmentation. (3) It is important to note that our analysis was limited to power spectral density metrics, which, while effective for capturing tonic, sustained spectral differences, do not capture the temporal dynamics of neural oscillations (e.g., burst duration, amplitude fluctuations, or instantaneous phase-locking). Therefore, our study cannot speak to whether the neural activity in the MDS group is characterized by greater instability or aberrant functional connectivity on a moment-to-moment basis, which represents an important method for future research. Furthermore, using specific resting EEG indicators to identify mild depressive symptoms in college students represents a preliminary attempt. These indicators may hold promise as objective markers for future auxiliary screening in this population. Further research is needed to validate these findings, and larger sample sizes and external validation are required to develop a mature resting EEG identification model. (4) This cross-sectional study cannot establish causality. We can only explore correlations between variables. Future research may consider conducting prospective cohort studies or randomized controlled trials to confirm the causal relationship between the aforementioned indicators and mild depressive symptoms.

## Conclusion

Our findings indicate that college students with mild depressive symptoms (MDS) may exhibit distinct behavioral patterns and neurophysiological characteristics compared to those without depressive symptoms (ND). Specifically, the group with with mild depressive symptoms exhibited reduced Moderate-to-Vigorous Physical Activity (MVPA) and increased Sedentary Behavior (SB) and Poor Sleep Quality (PSQ). At the neurophysiological level, the group with mild depressive symptoms exhibited elevated α2 power in the temporal regions (T3 and T5) and increased β1 power in the frontal region (Fp1), suggesting potential alterations in neural activity associated with arousal and emotional regulation. Additionally, multiple EEG indicators showed trends of intergroup differences before correction, which shows their potential as neuro-physiological markers. This warrants further validation in larger future samples. Exploratory analyses further revealed that sedentary behaviors and poor sleep quality were positively correlated with mild depressive symptoms, while Moderate-to-Vigorous Physical Activity were negatively correlated with mild depressive symptoms. Additionally, the combination of certain resting EEG indicators (α2 at T3 and T5, β1 at Fp1) demonstrated moderate discriminatory ability in identifying mild depressive symptoms, supporting the potential utility of resting EEG as a future objective measurement tool. Notably, within the MDS group, we observed correlations between sleep parameters and resting EEG specificity, suggesting a potential link between sleep and neurophysiological function in individuals with mild depressive symptoms. This study links behavioral patterns and resting EEG characteristics among college students to mild depressive symptoms, attempting to integrate the potential value of multidimensional exploration. This may contribute to a more nuanced understanding of the development of mild depressive symptoms and inform targeted interventions aimed at improving health behaviors and monitoring neurobiological correlates. However, due to the limitations of cross-sectional designs, this evidence cannot support causal inferences.

## Data Availability

The original contributions presented in the study are included in the article/supplementary material, further inquiries can be directed to the corresponding author.

## Abbreviations

MDS: Mild depressive symptoms
ND: Non-depressive symptoms
PA: Physical activity
VPA: Vigorous physical activity
MPA: Moderate physical activity
LPA: Low physical activity
MVPA: Moderate-to-Vigorous physical activity
ST: Sedentary time
SB: Sedentary behavior
PSQI: Pittsburgh Sleep Quality Index
PSQ: Poor Sleep Quality
Delta: δ
Theta: θ
Alpha1: α1
Alpha2: α2
Beta1: β1
Beta2: β2
